# Personal identification with orthopantomography using simple convolutional neural networks: a preliminary study

**DOI:** 10.1038/s41598-020-70474-4

**Published:** 2020-08-11

**Authors:** Shinpei Matsuda, Takashi Miyamoto, Hitoshi Yoshimura, Tatsuhito Hasegawa

**Affiliations:** 1 Department of Dentistry and Oral Surgery, Unit of Sensory and Locomotor Medicine, Division of Medicine, Faculty of Medical Sciences, University of Fukui, 23-3 Matsuokashimoaizuki, Eiheiji-cho, Yoshida-gun, Fukui 910-1193 Japan; 2 Graduate School of Engineering, University of Fukui, Fukui, Japan

**Keywords:** Engineering, Medical research

## Abstract

Forensic dental examination has played an important role in personal identification (PI). However, PI has essentially been based on traditional visual comparisons of ante- and postmortem dental records and radiographs, and there is no globally accepted PI method based on digital technology. Although many effective image recognition models have been developed, they have been underutilized in forensic odontology. The aim of this study was to verify the usefulness of PI with paired orthopantomographs obtained in a relatively short period using convolutional neural network (CNN) technologies. Thirty pairs of orthopantomographs obtained on different days were analyzed in terms of the accuracy of dental PI based on six well-known CNN architectures: VGG16, ResNet50, Inception-v3, InceptionResNet-v2, Xception, and MobileNet-v2. Each model was trained and tested using paired orthopantomographs, and pretraining and fine-tuning transfer learning methods were validated. Higher validation accuracy was achieved with fine-tuning than with pretraining, and each architecture showed a detection accuracy of 80.0% or more. The VGG16 model achieved the highest accuracy (100.0%) with pretraining and with fine-tuning. This study demonstrated the usefulness of CNN for PI using small numbers of orthopantomographic images, and it also showed that VGG16 was the most useful of the six tested CNN architectures.

## Introduction

Dental examination findings are unique among individuals because they consist of a combination of decayed, filled, prosthetic, and missing teeth^1^. Additionally, teeth are harder and more resistant to heat than other body tissues, including bones. Therefore, forensic dental examination has played an important role in personal identification (PI), even in large-scale accidents and disasters. Digitalization in medical and dental examinations and treatments has progressed, providing clinicians with great support in diagnoses and surgical procedures^2–4^. However, in forensic dental examination, PI has essentially been based on traditional visual comparisons of the antemortem dental records and radiographs with those obtained by postmortem dental examinations^5^. Moreover, such traditional forensic dental examinations have the disadvantage of lacking widely accepted reference points between antemortem records and postmortem examinations^1^. Finally, analyses of traditional written dental records are subjective^6^. Therefore, traditional forensic dental examinations are vulnerable to oversights and/or mistakes in PI^6^. The authors believe that one way to improve on these traditional dental examinations for dental PI that are currently used around the world would be to digitize and standardize them. Although forensic odontology with digital technologies has progressed, there is no globally accepted PI method based on digital technology due to various factors, such as special equipment and costs. With the progression of digital technologies, global digitization of forensic odontology should be promoted. In the future, such technologies will make a significant social contribution to PI in the wake of large-scale accidents and disasters.

In a position statement published by the Institute of Electrical and Electronics Engineers, the following definition of artificial intelligence (AI) is suggested: *AI is that activity devoted to making machines intelligent, and intelligence is that quality that enables an entity to function appropriately and with foresight in its environment*^7^. The development of AI technologies has made great strides along with the growth and evolution of personal computers. At present, there is a possibility that machines will displace humans in various fields, including medicine^8^. AI technology includes deep learning, which improves a machine’s ability to represent data by deepening the layered structure of neural networks; a convolutional neural network (CNN) is a type of network for deep learning^9–15^. The authors believe that CNNs may be useful for forensic PI because they have high recognition accuracy, mainly in the field of image recognition^9^. Thus, a convergence between dental imaging examination and CNNs in the forensic field may have great potential to bring social benefits by improving preparedness for large-scale accidents and disasters due to the frequency of imaging examinations in clinical dentistry.

The aim of this preliminary study was to evaluate the usefulness of PI with orthopantomography using the following six CNN architectures: VGG16, ResNet50, Inception-v3, InceptionResNet-v2, Xception, and MobileNet-v2^10–15^.

## Results

### Participants and materials

Thirty participants (14 male and 16 female) were included in this study (Table 1). Thus, 30 pairs of orthopantomographs, for a total of 60 orthopantomographs, were analyzed in this study. The youngest participant was 15 years old, and the oldest was 86 years old. The mean ± standard deviation was 31.00 ± 15.21 years old. Twenty-nine participants underwent orthopantomography before and after tooth extractions, and one participant underwent orthopantomography twice in follow-up examinations (Table 1: No. 11). The mean ± standard deviation of the number of extracted teeth among the thirty participants was 2.03 ± 1.07, and the most common extracted tooth was the mandibular third molar. The mean and standard deviation of the number of teeth “before orthopantomography” was 29.00 ± 6.34, and that “after orthopantomography” was 26.97 ± 6.08. The mean ± standard deviation of the interval between “before orthopantomography” and “after orthopantomography” was 64.03 ± 70.29 days.

**Table 1:** Characteristics of participants in the study.

No	Gender	Age	^a^Tooth extraction sites	Number of tooth extractions	Number of teeth before extraction	Number of teeth after extraction	Interval between initial and final panoramic radiography (days)
1	F	33	38	1	28	27	34
2	F	29	38, 48	2	30	28	49
3	M	28	38, 48	2	30	28	20
4	F	32	18, 28, 38, 48	4	32	28	309
5	F	25	18, 28, 38, 48	4	32	28	59
6	F	29	38, 48	2	30	28	84
7	F	21	38, 48	2	32	30	14
8	M	18	38	1	32	31	5
9	M	24	38, 48	2	30	28	35
10	F	21	28	1	31	30	119
11	F	33	Not applicable	0	27	27	199
12	F	33	38, 48	2	29	27	41
13	M	15	38, 48	2	32	30	78
14	F	36	28, 38, 48	3	31	28	49
15	M	27	18, 48	2	31	29	35
16	M	25	28, 38, 48	3	31	28	89
17	F	63	48	1	13	12	32
18	F	20	18, 28, 38, 48	4	30	26	149
19	F	28	38	1	31	30	21
20	F	24	38, 48	2	32	30	21
21	M	86	38	1	1	0	188
22	M	29	38	1	32	31	7
23	M	27	48	1	30	29	13
24	M	34	18, 17, 27	3	31	28	4
25	M	25	18, 38, 48	3	31	28	40
26	F	64	38	1	30	29	15
27	F	18	38, 48	2	32	30	30
28	M	42	18, 28, 38, 48	4	31	27	134
29	M	21	38, 48	2	31	29	36
30	M	20	38, 48	2	27	25	12
Average		31.00		2.03	29.00	26.97	64.03
Standard deviation		15.21		1.07	6.34	6.08	70.29

^a^Tooth numbering system proposed by the Fédération dentaire internationale (FDI).

### Training and testing a CNN model

In the training phase, the CNN used a training dataset composed of input images ***X*** and their correct personal identifiers ***y*** to adapt the weights (filters) of the network. After training the model, the CNN estimated the correct label $\widehat{y}$ from unlabeled image *X′* in the testing phase. Finally, the estimated label $\widehat{y}$ and the correct label y were compared to calculate evaluation indexes such as accuracy, precision, and recall. In this study, the authors used accuracy as an evaluation index.

The authors identified a suitable CNN architecture and training parameters for this task through comparison experiments. Each model was trained using the “before orthopantomography” dataset for 300 epochs and tested using the “after orthopantomography” dataset. In this study, the authors validated six types of CNN architecture: VGG16, ResNet50, Inception-v3, InceptionResNet-v2, Xception, and MobileNet-v2. These architectures were implemented in Keras (https://keras.io/), which is a Python deep learning library. The parameters and original papers are found in the official Keras documentation (https://keras.io/applications/#applications). The authors also validated some optimizers: SGD, RMSProp, Adadelta, Adagrad, and Adam, with a learning rate of 10^−5^ to 10^−3^. The batch size was 10. Furthermore, the authors validated two types of transfer learning methods: pretraining and fine-tuning. In pretraining, all weights in the model were trained in advance using a large-scale dataset for other tasks, and some of the weights were trained using the target dataset (e.g., the dental PI dataset in this study). The authors trained only the weights of the final layer after pretraining. In fine-tuning, all weights in the model were retrained using the target dataset after pretraining. Both transfer learning methods used the ImageNet dataset, which is a very large-scale image recognition dataset including over 14 million labeled images. In total, the authors validated 180 training patterns (6 models × 5 optimizers × 3 learning rates × 2 transfer methods).

### Evaluation results

**Figure 1:**
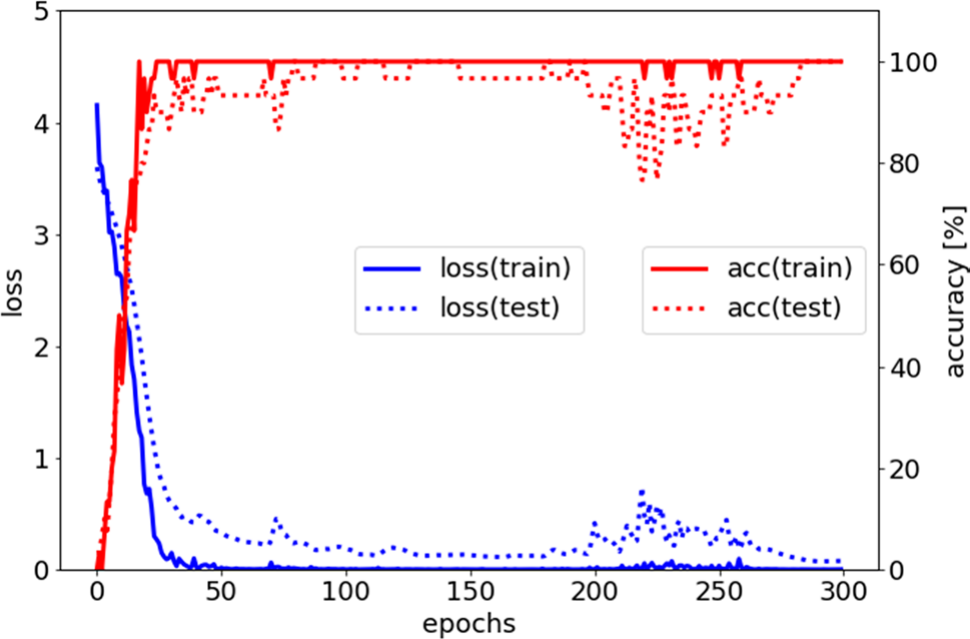
The loss (blue line) and accuracy (red line) in training. The x-axis measures epochs, the left y-axis measures loss, and the right y-axis measures accuracy (%). Focusing on the red line, the training and testing accuracies converge after 30 epochs. Although the training data are perfectly classified, the testing data are sometimes misclassified.

The values in the table represent the validation accuracies in the final epoch (Table 2). The authors selected the maximum accuracies from all optimizers for each method because the best optimizer and learning rate differ for each CNN architecture. In this evaluation, the VGG16 model achieved the highest accuracy, at 100.0% (Fig. 1). When drawing comparisons in transfer learning, the most accurate method to use is fine-tuning. Under fine-tuning, some CNN architectures also achieved greater than 90.0% accuracy. VGG16, despite having a relatively shallow layer structure, had the highest estimation accuracy because this dataset was very small.

**Table 2:** Validation accuracies for each CNN architecture and transfer learning method.

	VGG16 (%)	ResNet50 (%)	Inception-v3 (%)	Inception ResNet-v2 (%)	Xception (%)	MobileNet-v2 (%)
Pretraining	100.0	3.3	20.0	26.7	20.0	16.7
Fine-tuning	100.0	93.3	83.3	96.7	80.0	80.0

## Discussion

In this study, the authors evaluated the usefulness of the application of CNN technology in PI based on orthopantomography by validating the identification accuracy of six different CNN architectures. The results demonstrated that VGG16 was the most useful CNN architecture for PI using orthopantomography. Furthermore, VGG16 pretraining and fine-tuning using the ImageNet dataset achieved 100% identification accuracy. In cases of forensic medicine and forensic dentistry such as fire and murder victims, excluding large-scale disasters, it is important to identify individuals from a small number of people. To resolve this problem, this study indicated the possibility of accurate PI from orthopantomography image by using CNNs.

According to the PubMed database, studies of CNN application have been reported since 2013 in various medical including orthopedics, oncology, ophthalmology, and neurosurgery^16–19^. In the dental field, Miki et al. first reported the application of CNNs with cone-beam computed tomography in 2017^20^. In recent years, applications of CNNs in cariology, periodontology, and endodontics have been reported, and applications in real dental clinical experience may be realized in the near future^21–23^. Tuzoff et al. reported a method of tooth detection and numbering with orthopantomography using simple CNNs, which could help save time and improve the process of filling out dental charts^24^. Kahaki et al. tried to establish an age estimation method based on global fuzzy segmentation and local feature extraction using a projection-based feature transform and a designed deep CNN model; the molars were isolated from 456 young participants' orthopantomography as an analysis target^25^. Schwendicke et al. performed a scoping review of CNNs for dental image diagnostics and concluded that CNNs may be used in diagnostic-assistance systems in the dental field^26^. At present, CNN technology is not easy for dentists to implement. In the future, generalization of such technology will further advance through the development of simple software.

As a limitation of this study, the authors must note that the mean ± standard deviation of the periods between the date obtained “before orthopantomography” and the date obtained “after orthopantomography” was 64.03 ± 70.29 days; in other words, the paired orthopantomographs under comparison were obtained at relatively short intervals. This condition was due to the inclusion criteria for the study participants to rule out age-related oral changes and oral diseases such as periodontal disease and caries. Twenty-nine of the 30 participants were examined orthopantomographically before and after wisdom tooth extraction, while one participant did not undergo tooth extraction during the study. The authors considered that the reason why the VGG16 model achieved 100.0% accuracy was because the images taken as “before orthopantomography” and “after orthopantomography” were obtained under almost the same conditions and were very similar. Therefore, the results of this preliminary experiment verified the applicability of PI in an ideal environment with pairs of very similar images indicated that CNNs can classify individual orthopantomographs according to which patients they depict. In addition, because the authors were able to use only two datasets at this time, they established only a training set and a validation set. The authors have been able to perform only limited verification. Normally, it is necessary to prepare the test data. Additionally, it is necessary to train the model with a training set, perform parameter tuning with a validation set, and then verify the final estimation accuracy with a test set. This is one of the limitations of this study. In addition, the authors understand that the application of deep neural networks to small datasets may lead to the problem of overfitting. On the other hand, there are several issues with long-term changes, including periodontal bone loss, dental caries, and prosthetic rehabilitation, in studies of PI based on orthopantomography, as mentioned above. Additionally, experimental orthopantomography examination will lead to radiation exposure and ethical issues. In this study, participants who underwent orthopantomography for clinical treatment in a relatively short period were collected retrospectively and analyzed. Therefore, it was difficult to construct a large-scale image dataset. To use CNN technology for orthopantomography in preparation for large-scale accidents and disasters in the future, further validation of the number of paired orthopantomographs and the interval periods between “before orthopantomography” and “after orthopantomography” is required. Additionally, the main cause of death might be facial trauma with tooth or bone fractures. Therefore, various clinical situations such as changes in maxillofacial and dental status should be considered in PI, and PI should be performed with high accuracy. Based on this preliminary study, it is important to proceed with PI research using CNN technology but also considering the cause of death in order to apply the results of this study to actual forensic cases in the future. Regarding the loss function, the best-known loss function for classification tasks is cross-entropy loss, which is defined by the equation in this study. Mean squared error and mean absolute error can also be used as loss functions in classification, and there are other loss functions as well, such as focal loss, class-balanced loss, and triplet loss. Because this experiment was a preliminary study, the authors adopted the best-known loss function: cross-entropy loss.

Generally, orthopantomography requires standing and/or proper posture. Therefore, obtaining postmortem orthopantomographs may be difficult. Franco et al. suggested that postmortem full-body CT images could become a valuable tool in individual dental identification procedures^27^. Ohtani et al. reported radiographic imaging examination using portable devices acceptable for prone position^28^. In the future, the authors suggest that dentists and engineers should consider methods of constructing an orthopantomography-like image that is acceptable for postmortem examination by using data from another modality, such as CT images; CNN technology could then be applied for PI with those images.

In conclusion, this study demonstrated the usefulness of CNN for PI using a small number of orthopantomographic images and that VGG16 was more useful than five other CNN architectures. Further validation in the number of paired images and in the interval between “before orthopantomography” and “after orthopantomography” is required to use this technology for large-scale accidents and disasters in the future.

## Materials and methods

### Participants and materials

**Figure 2:**
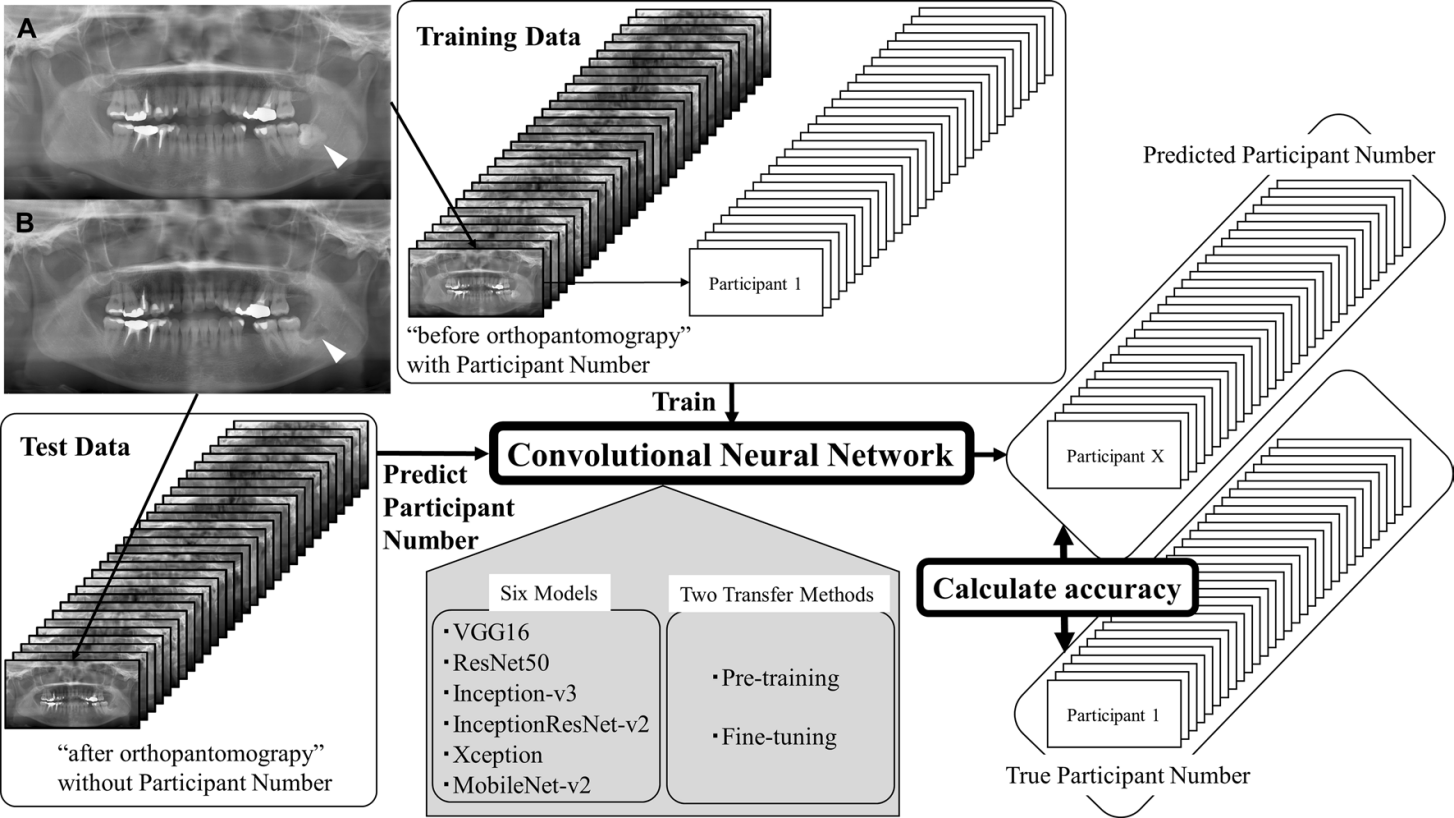
The design of this study was based on paired orthopantomographs analyzed with six convolutional neural network architectures. The paired orthopantomographs of participant No. 1 are shown as (**A**) “before orthopantomography” and (**B**) “after orthopantomography”, and tooth extraction sites are indicated by white arrows in this figure.

The authors designed a retrospective study. The participants were patients who received orthopantomography for clinical treatment at the University of Fukui Hospital twice from January 2018 to September 2019 and at relatively short intervals to rule out age-related oral changes and oral diseases such as periodontal disease and caries. Paired orthopantomographs of those participants were analyzed for the development of the PI method. In this study, based on the examination date, paired orthopantomographs were defined as “before orthopantomography” and “after orthopantomography” (Fig. 2). The orthopantomography data used in this study are stored in the Image Management System of the University of Fukui Hospital, and the details of the participants are shown in Table 1.

### Simple convolutional neural networks

Machine learning is a method to explore a function f(⋅) that can convert x to y from a dataset consisting of input data x with the correct label y. The goal is to optimize the formula y = f(x) from a given dataset. A CNN is a neural network model composed of many layers, including a convolution layer and a pooling layer. CNNs are aggressively applied in the image recognition field—for example, cancer detection from X-ray images. The basic principle of the convolution process is to calculate the convolution from a raw image with filters. Filters, which are similar to weights in a neural network, are adoptively adjusted in the training process. By this process, a computer aims to acquire the effective filters to detect the target from the dataset. The basic principle of the pooling process is to calculate the representative value from fixed-size windows—for example, a 2 × 2 max pooling layer calculates the max values from each 2 × 2 array of pixels. Through this process, it is said that a computer acquires a robust feature representation of the target position.

### Formulation of this problem

The authors formulated a dental PI task to complete via a CNN model. In this task, a dental image is given, and the personal identifier is required as an output. The input for machine learning is a dental image *X*, and the output is a personal identifier y. Therefore, CNN model *f(*⋅*)* takes the role of a converter that converts input image X into personal identifier *y*. This is a simple classification task represented by the formula *y* = *f(X)*.

In order to accurately estimate the personal identifier y, CNN filters are adapted based on the loss function. There are many CNN architectures and loss functions in the facial recognition field; however, as a preliminary experiment, this study adopted a simple CNN classifier with a common loss function called categorical cross-entropy loss (*L*_*ce*_). *L*_*ce*_ is represented by the following formula:

$${L}_{ce}=-\sum_{i}^{N}{t}_{i}{\text{log}}\left\{{f(X)}_{i}\right\}$$

where *N* is the number of target people, *t* is a correct label (one-hot vector), and *f(X)*_*i*_ is the i-th CNN’s output. CNN adopts its filters to minimize *L*_*ce*_ in the training phase.

### Ethics approval and informed consent

This study was approved by the Institutional Research Board (Ethical Committee of the University of Fukui, Faculty of Medical Sciences, No. 20190043). There were no ethical issues in conducting this study because it was a retrospective study targeting examination images obtained for clinical treatment, and the authors declared that all methods were performed in accordance with the relevant guidelines and regulations (Declaration of Helsinki). Additionally, informed consent was obtained from all subjects or, if subjects were under 18, from a parent and/or legal guardian.

## Data Availability

The data used to support the findings of this study are available from the corresponding author upon request.
